# Genome-wide screening for differentially methylated long noncoding RNAs identifies LIFR-AS1 as an epigenetically regulated lncRNA that inhibits the progression of colorectal cancer

**DOI:** 10.1186/s13148-022-01361-0

**Published:** 2022-10-31

**Authors:** Peng Song, Ying Li, Feng Wang, Lingxiao Pu, Linsen Bao, Hengfei Gao, Chuandong Zhu, Meng Wang, Liang Tao

**Affiliations:** 1 Department of Gastrointestinal Surgery, Nanjing Drum Tower Hospital, The Affiliated Hospital of Nanjing University Medical School, Nanjing, China; 2 State Key Laboratory of Natural Medicines, School of Traditional Chinese Pharmacy, China Pharmaceutical University, Nanjing, China; 3 Department of Oncology, Nanjing Second Hospital, Nanjing University of Chinese Medicine, Nanjing, China

**Keywords:** LIFR-AS1, Methylation markers, Prognosis, Colorectal cancer

## Abstract

**Background:**

Aberrant DNA methylation is an epigenetic marker that has been linked to the pathogenesis of colorectal cancer (CRC). Long noncoding RNAs (lncRNAs) have been increasingly identified to be associated with tumorigenic processes of CRC. Identifying epigenetically dysregulated lncRNAs and characterizing their effects during carcinogenesis are focuses of cancer research.

**Methods:**

Differentially methylated loci and expressed lncRNAs were identified by integrating DNA methylome and transcriptome analyses using The Cancer Genome Atlas database. Bisulfite sequencing PCR (BSP) was performed to analyze LIFR-AS1 promoter methylation status. The functional roles of LIFR-AS1 in CRC were determined by in vitro and in vivo experiments.

**Results:**

We identified a novel hypermethylated lncRNA, LIFR-AS1, that was downregulated and associated with tumorigenesis, metastasis, and poor prognosis in CRC. High methylation burden of LIFR-AS1 indicated a poor survival of CRC patients. Promoter hypermethylation of LIFR-AS1 in tumor tissues was confirmed by BSP. Functional assays revealed that LIFR-AS1 could competitively bind to hsa-miR-29b-3p, and repressed colon cancer cell proliferation, colony formation and invasion. LIFR-AS1 also inhibited tumor growth in a mouse xenograft model of CRC.

**Conclusions:**

Our results showed that the identified DNA methylation-dysregulated lncRNAs may be potential biomarkers and highlighted a role for LIFR-AS1 as a tumor suppressor in CRC.

**Supplementary Information:**

The online version contains supplementary material available at 10.1186/s13148-022-01361-0.

## Introduction

Colorectal cancer (CRC) is the third most common malignancy and the second highest cancer-related cause of death in the world [1]. CRC is caused by the accumulation of multiple genetic and epigenetic alterations in the genome [2, 3]. Over the past decade, research has focused on better understanding of cancer epigenetics, particularly regarding aberrant DNA methylation, microRNA and long noncoding RNA (lncRNA) deregulation, to identify prognostic and predictive factors for cancer [3]. DNA methylation is a reversible and regulatory modification, and changes in DNA methylation can occur in the early stage of cancer development. DNA methylation has also been shown to be a candidate biomarker in cancer [4]. For example, *SEPT9* gene methylation has been implicated as a biomarker for predicting CRC [5]. Methylated *SEPT9* gene in serum is closely related to the advanced stage of CRC [6]. Increasing studies have revealed cancer-linked aberrant methylation of protein-coding gene promoters. However, the genome-wide identification of differential DNA methylation and expression of lncRNAs with functional importance in CRC is still lacking.

LncRNAs are crucial regulators at the transcriptional and post-transcriptional levels and are involved in diverse biological functions [7]. Aberrant lncRNA expression in cancers can be caused by alteration of epigenetic patterns, such as changes in DNA methylation. For instance, Lin et al. reported that lncRNA DLX6-AS1 hypermethylation was present in colorectal neoplasms at all stages and increased during colorectal carcinogenesis [8]. Hypermethylation of DLX6-AS1 was also detected in cell-free DNA samples from CRC patients. Mamivand et al. identified an epigenetically deregulated lncRNA OBI1-AS1 with decreased expression in glioblastoma multiforme. Hi-C and ChIP-Seq analysis showed that methylation of the CTCF binding site blocked the expression of OBI1-AS1 by influencing chromatin interactions [9]. LncRNAs also function as a scaffold for the recruitment of chromatin modifiers to target promoters. In CRC cells, linc00337 recruited DNA methyltransferase 1 (DNMT1) to the promoter region of *CNN1* and restricted its transcription, promoting tumor growth and angiogenesis [10]. DNA methylation aberrations in cancer and the crosstalk with lncRNAs are research hotspots. The availability of high-throughput sequencing technology has facilitated the exploration of epigenetic changes across the genome. Therefore, here we used a reannotation strategy to construct the DNA methylation profile of lncRNAs in CRC. In this study, we screened and identified methylation-driven differentially expressed lncRNAs in CRC, with the aim of improving diagnosis and personalized treatment of CRC patients.

## Methods

### Data and patient selection

The DNA methylation array data (Illumina Infinium Human Methylation 450 BeadChip) and level 3 RNA-sequencing data (HTSeq-Counts and HTSeq-FPKM-UQ) along with clinicopathological information were downloaded from the UCSC Xena browser (https://xenabrowser.net/). Level 3 miRNA-seq data were obtained through TCGA Genomic Data Commons portal (GDC). We also downloaded RNA sequencing data of CRC from the Gene Expression Omnibus (GEO, www.ncbi.nlm.nih.gov/geo) database (GSE156451). An in-house dataset including 92 CRC tissues and 43 normal tissues from patients who underwent surgery at Nanjing Drum Tower Hospital (The Affiliated Hospital of Nanjing University Medical School, Nanjing, China) was also used for analysis. All patients were pathologically diagnosed with colon adenocarcinoma following the American Joint Committee on Cancer’s criteria. None of the patients received preoperative chemotherapy or radiotherapy. The adjacent normal tissues were collected > 5 cm from the tumor margins. All patients provided written informed consent, and this study was approved by the Research Ethics Committee of Nanjing Drum Tower Hospital.

### Integrated analysis of DNA methylation and lncRNA expression

To identify differentially expressed lncRNAs, RNA-seq read count tables mapped on the hg38 human genome with GENCODE v22 as gene annotation were imported into three statistically-based expression analysis tools (edgeR [11], limma [12], and DESeq2 [13]). Differentially methylated CpG sites between CRC samples and adjacent tissues were identified using the “minfi” package. Conjoint analysis of the methylome and transcriptome of lncRNAs was performed as previously described [14]. Briefly, the genomic coordinates of each CpG site and individual lncRNA were extracted from hm450.hg38.manifest and GENCODE v22 files, respectively [14]. We combined both information using the above genomic location, taking the differentially methylated loci within promoter regions (DNA sequences between −2500 and 1000 bp relative to the putative transcription start site) into account.

### Bisulfite sequencing PCR (BSP)

Cellular genomic DNA was isolated from fresh frozen tissues using the Genomic DNA extraction kit (TIANamp Genomic DNA Kit, DP304). The purified DNA was bisulfite-treated using the EpiTect Fast DNA Bisulfite Kit (Qiagen) following the manufacturer’s protocol. The bisulfite-modified promoter regions were amplified by BSP primers (forward primer: GGAGGAAAAATTTTATTTTATTAAGA, reverse primer: ACCRAACCCAAACAAATCCTC). Amplified sequences were cloned into the pMD18-T vector and sequenced. The sequencing results were compared to the original sequence using the QUMA website (http://quma.cdb.riken.jp/). To determine the methylation rate, 10 clones were required for each sample.

### Reverse transcription quantitative polymerase chain reaction (RT-qPCR)

Total RNA was extracted from tissues or cells using TRIzol reagent (Invitrogen, USA), and reverse transcription was conducted with the Primescript RT Reagent Kit (TaKaRa, Japan) following company protocols. RT-qPCR was performed on the 7900 Real-Time PCR System (Applied Biosystems, USA) using the SYBR Premix Ex Taq Kit (TaKaRa, China). The primers used for amplification were as follows: F: 5′- AAGTTTCAGGCTCCTGACAGC -3′ and R: 5′- TTCGCCCACGTTCTTCTCGC -3′ for LIFR-AS1, F: 5′- TGGAACGACAGGGGTTCAGT -3′ and R: 5′- GAGTTGTGTTGTGGGTCACTAA -3′ for LIFR, and F: 5′- AGAAGGCTGGGGCTCATTTG -3′ and R: 5′- AGGGGCCATCCACAGTCTTC -3′ for GAPDH.

### Cell culture and construction of stable cell lines

The human colon cancer cell lines LOVO, HCT116, SW480, SW620, DLD-1, HT-29 and T84 were purchased from the Chinese Academy of Sciences, China. Cells were cultured in RPMI 1640 medium (Gibco, USA), and supplemented with 10% fetal bovine serum (FBS) and 1% penicillin/streptomycin. For overexpression of LIFR-AS1, the sequence of LIFR-AS1 was synthesized and subcloned into the pcDNA3.1 plasmid. HT-29 and T84 cells were transfected with the LIFR-AS1 expression plasmid (oe-LIFR-AS1) or the empty vector (NC) as control using Lipofectamine 3000 (Invitrogen, USA). Overexpression was evaluated by RT-qPCR.

### Luciferase reporter gene assay

Amplification of cDNA fragments of LIFR-AS1 wild type (WT) and mutant (MUT) containing binding sites of hsa-miR-29b-3p were cloned into a psiCHECK-2 vector (Promega, USA). The LIFR-AS1_WT vector or LIFR-AS1_MUT vector and hsa-miR-29b-3p mimics were co-transfected into HT-29 and T84 cells by Lipofectamine 3000 (Invitrogen, USA). After 24 h of culture, the luciferase intensity was assessed by Promega Dual-Luciferase Reporter Assay System.

### Colony formation, cell viability and invasion assays

For colony formation assays, approximately 1 × 10^3^ cells in DMEM medium supplemented with 10% FBS were cultured at 37 °C and 5% CO_2_ for two weeks. Cell colonies were photographed and counted.

For CCK-8 assays, transfected cells were plated into 96-well plates (approximately 1 × 10^4^/well) and incubated with CCK-8 solution (Dojindo, Japan) for 1 h at 37 °C. The optical density value at 450 nm was measured.

For invasion assays, cells at a density of 1 × 10^5^ cells/ml were seeded in the upper chambers of 24-well transwell systems (Corning, USA) coated with a polycarbonate membrane, and 600 ml DMEM medium containing 15% FBS was added into the lower chamber. Cells were cultured for 24 h. After removing non-invading cells, the remaining cells were fixed with 4% paraformaldehyde and stained with 0.1% crystal violet (Beyotime, China) at room temperature for 30 min. The invaded cells were counted under five randomly selected views using a phase-contrast microscope (Nikon, Tokyo, Japan).

### Xenograft tumor model

Transfected HT-29 cells (1 × 10^7^/ml) were injected into 6-week-old female BALB/C nude mice. Tumor growth (determined by measuring length and width) was monitored and recorded. Tumor volume was calculated by the formula (length × width^2^ × 1/2). The mice were euthanized after 4 weeks, and the tumors were extracted and analyzed. This study was approved by the Animal Ethics Committee of Experimental Animal Center of Nanjing Drum Tower Hospital.

### Bioinformatics analysis

The R package ClusterProfiler was used to perform Gene Set Enrichment Analysis (GSEA) and plot the results [15]. The input data was ranking metric using the value of log2 (Fold Change) calculated by “DESeq2” package. The reference gene sets were retrieved from the C2 collection in the Molecular Signatures Database. Single-cell RNA-sequencing (scRNA-seq) data for CRC (GSE144735) were obtained from the GEO database [16]. Seurat package was used for downstream analysis, and the t-SNE algorithm was used for nonlinear dimension reduction of the scRNA-seq data [17].

### Statistical analysis

The Student’s *t* test or Mann–Whitney test (for continuous variables) and Pearson’s *χ*^2^ test (for categorical variables) were used to examine differences between two groups. Kaplan–Meier method and log-rank test were employed to evaluate gene expression or methylation level on the survival of patients. Multivariate Cox regression analysis was used to estimate adjusted hazard ratios and 95% confidence intervals for LIFR-AS1 expression. All statistical analysis was conducted on R programming language v4.1.2, and two-sided and *P* < 0.05 was defined as statistically significant.

## Results

### Integrative analysis of differential methylation and lncRNA expression data in CRC

**Fig. 1:**
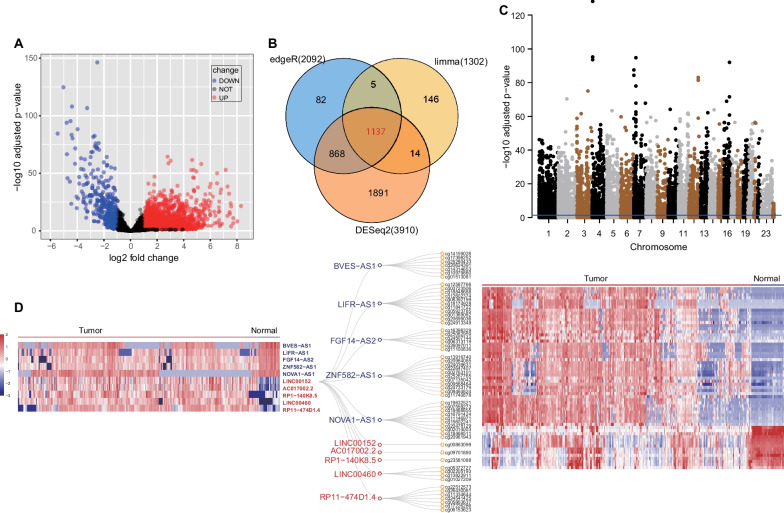
Conjoint analysis of the methylome and transcriptome of lncRNAs in CRC. **A** Volcano plot of differentially expressed lncRNAs between tumors and normal tissues. **B** Venn diagram displaying the overlap of lncRNAs identified using three algorithms (edgeR, limma and DESeq2). **C** Manhattan plot of CpG sites in the promoter regions of lncRNA genes; dots above the blue line indicate CpG sites with *P* value < 0.05. **D** Heatmap of top five up- and downregulated lncRNAs between tumors and normal tissues and the hierarchical clustering heat map of 10 lncRNA-related CpG sites. Color scale represents the relative expression level or methylation level for each lncRNA

We first performed differential expression analysis for lncRNAs using RNA-seq data of 471 primary colon tumors and 41 normal tissues from The Cancer Genome Atlas (TCGA). Using three algorithms (edgeR, limma and DESeq2), a total of 1137 lncRNAs (825 upregulated lncRNAs and 312 downregulated lncRNAs) were identified using the criteria of false discovery rate (FDR) < 0.05 and absolute fold change (FC) ≥ 2 (Fig. 1A, B). Long intergenic noncoding RNAs (lincRNAs) accounted for most (45.03%) of all differentially expressed lncRNAs, followed by antisense transcripts (36.59%) (Additional file 1: Fig. S1).

To explore the DNA methylation pattern of lncRNAs in CRC, we then compared the differentially methylated CpG sites in 309 tumor and 38 normal tissues. A total of 16,266 CpG sites with FDR < 0.05 were obtained in the promoters of lncRNAs (Fig. 1C). We further identified 432 differentially methylated regions from the following parameters: resamples = 100, cut-off = 0.2, and probe number ≥ 2. These two omics data (methylome and transcriptome) were combined for further analysis. By associating the 16,266 CpG sites to 1137 lncRNAs, 276 pairs of methylation-driven lncRNAs were identified (Additional file 2: Table S1). The top five hypermethylated lncRNAs (BVES-AS1, ZNF582-AS1, FGF14-AS2, NOVA1-AS1 and LIFR-AS1) and top five hypomethylated lncRNAs (AC017002.2, LINC00152, RP1-140K8.5, LINC00460 and RP11-474D1.4) in CRC are shown in Fig. 1D.

### Downregulation of LIFR-AS1 in CRC and its functional characteristics

**Fig. 2:**
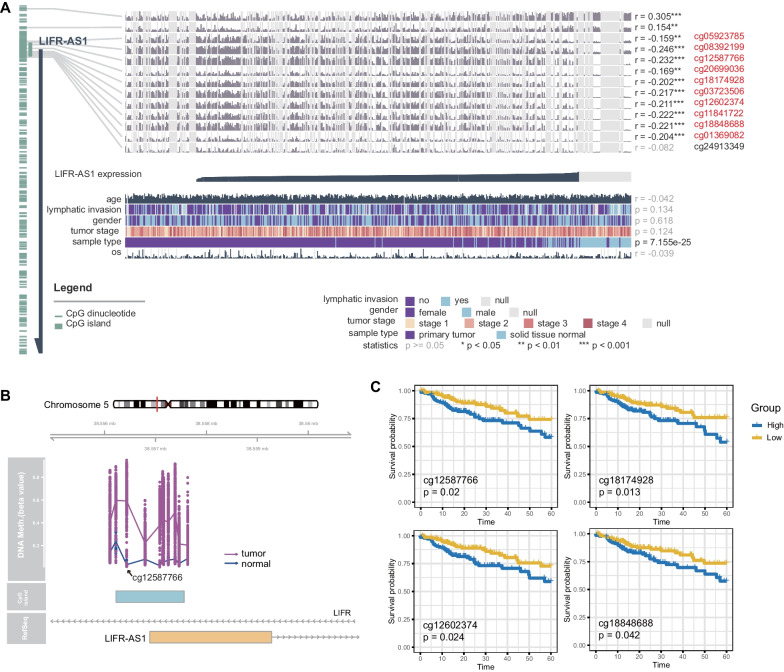
Association of LIFR-AS1 methylation and clinical characteristics in CRC using TCGA database. **A** The relationship between LIFR-AS1 methylation and its expression using MEXPRESS. **B** LIFR-AS1 promoter hypermethylation in CRC tumors compared with normal tissues. **C** Kaplan–Meier analysis of the overall 5-year relative survival rate for CRC patients according to the methylation levels of cg12587766, cg18174928, cg12602374 and cg18848688

The highest levels of methylation were observed in LIFR-AS1. Therefore, we performed further analysis to explore its biological function. Ten CpG sites in the LIFR-AS1 promoter (cg05923785, cg08392199, cg12587766, cg20699036, cg18174928, cg03723506, cg12602374, cg11841722, cg18848688 and cg01369082) were significantly correlated with LIFR-AS1 expression via MEXPRESS (correlation coefficients from − 0.159 to − 0.246, Fig. 2A, Additional file 3: Fig. S2). Colon tumors exhibited significantly higher levels of hypermethylation compared with normal tissues (Fig. 2B). Among the above 10 CpG sites, cg12587766 showed prominent methylation with a mean delta beta value of 0.559. Kaplan–Meier curves showed that four CpG sites (cg12587766, cg18174928, cg12602374, and cg18848688) were associated with the overall 5-year relative survival rate of CRC patients, which indicated that high methylation burden group had significantly poor survival (Fig. 2C).

**Fig. 3:**
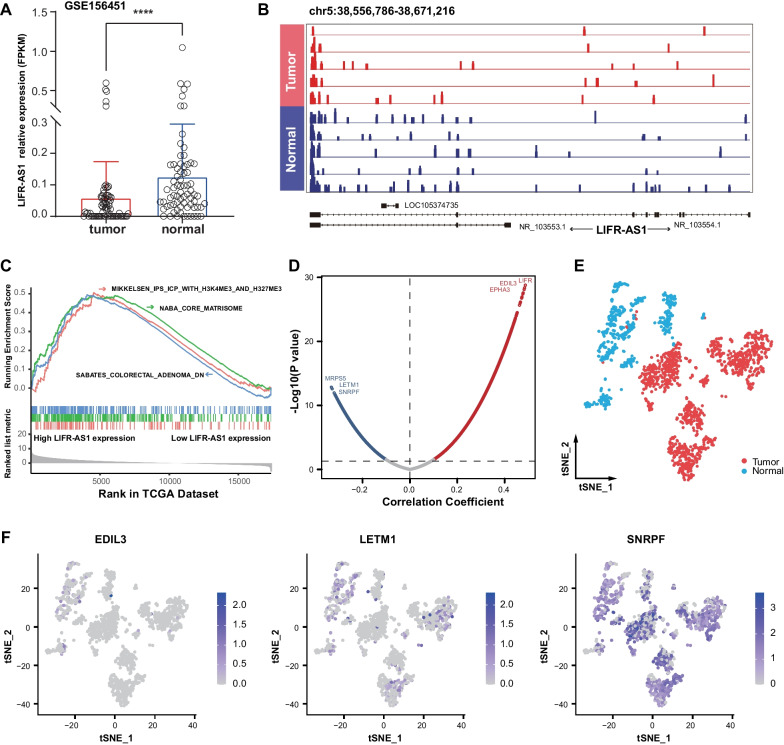
Functional annotation for the differentially expressed lncRNA LIFR-AS1. **A** The expression of LIFR-AS1 between CRC tumors and normal tissues in the GSE156451 dataset. **B** Representative RNA-sequencing results of mapped reads for two LIFR-AS1 isoforms in the GSE156451 dataset. **C** Gene set enrichment analysis of LIFR-AS1 in The Cancer Genome Atlas (TCGA) dataset. **D** Volcano map shows the co-expression genes associated with LIFR-AS1 expression in TCGA dataset. **E** The t-distributed stochastic neighbor embedding (t-SNE) plot of epithelial cells from CRC in the KUL3 dataset. **F** The t-SNE plots color-coded (gray to blue) to represent the expression levels of EDIL3, LETM1 and SNRPF

LIFR-AS1 is located on chromosome 5 (38,556,786–38,671,216) and encodes two different transcripts: NR_103554.1 is 3386 bp in length with nine exons and NR_103553.1 is 3803 bp in length and contains three exons. RNA-seq is widely used for transcript quantification of gene isoforms. We used RNA expression data available from GSE156451 to identify the products transcribed from this locus in CRC [18]. Consistent with our previous results, the expression of LIFR-AS1 was downregulated in CRC (Fig. 3A). As shown in Fig. 3B, most aligned reads were mapped within the exons in NR_103554.1. We thus chose this sequence for further analysis.

We ranked 18,524 genes from CRC samples in the TCGA dataset by their relative LIFR-AS1 expression in the top 10th percentile vs. the bottom 10th percentile for GSEA. The “MIKKELSEN_IPS_ICP_WITH_H3K4ME3_AND_H327ME3”, “NABA_CORE_MATRISOME” and “SABATES_COLORECTAL_ADENOMA_DN” sets were enriched in the LIFR-AS1 high expression group, which implied that this lncRNA might be involved in colorectal carcinogenesis (Fig. 3C). Using the threshold selection of absolute correlation coefficient > 0.1 and *P* value < 0.05, we found that 7681 genes were positively correlated and 3387 genes were negatively correlated with the expression of LIFR-AS1 (Fig. 3D). As scRNA-seq technology could not detect the expression of LIFR-AS1, we then investigated the expression of LIFR-AS1-related genes (*LIFR*, *EDIL3*, *EPHA3*, *MRPS5*, *LETM1* and *SNRPF*) in 1678 epithelial cells from CRC (Fig. 3E). Interestingly, despite a low level of expression overall, *EDIL3* was highly expressed in normal epithelial cells compared with CRC cells. In addition, elevated levels of *LETM1* and *SNRPF* were detected in malignant epithelial cells (Fig. 3F).

**Fig. 4:**
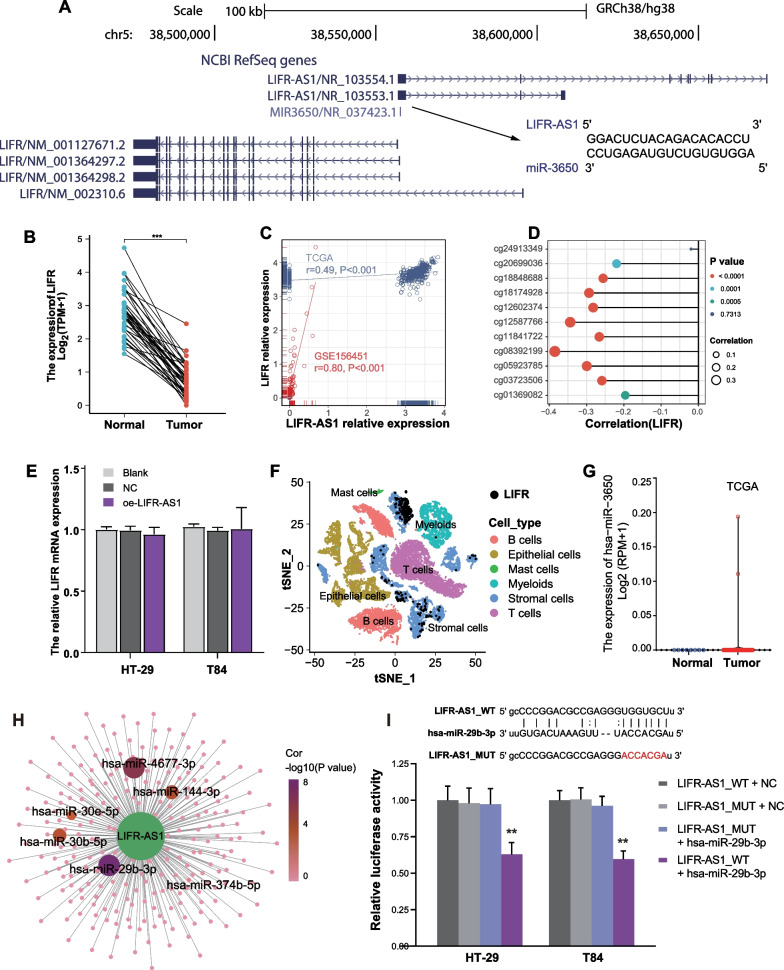
The features of LIFR-AS1 and neighboring genes in CRC. **A** The position of LIFR-AS1 and neighboring genes (LIFR and miR-3650) annotated in GRCh38. **B** The expression of *LIFR* in 41 matched normal and tumor CRC samples from TCGA. **C** Correlation analysis between LIFR-AS1 and *LIFR* expression in GSE156451 and TCGA datasets. **D** Correlations between *LIFR* expression and methylation level of CpG loci located in LIFR-AS1 promoter region. **E** Expression levels of *LIFR* in LIFR-AS1 overexpression cells using qRT-PCR analysis. **F** The expression of *LIFR* in t-SNE plots colored by cell cluster. **G** The expression of miR-3650 in CRC samples from TCGA. **H** The transcriptional regulatory network showing the target genes of LIFR-AS1. Color represents the P value. **I** Luciferase activities of LIFR-AS1_WT and LIFR-AS1_MUT with or without transfection of hsa-miR-29b-3p

The LIFR-AS1 sequence overlaps with the *LIFR* gene (Fig. 4A). *LIFR* was downregulated in CRC tumors compared with paired normal tissues in TCGA dataset (Fig. 4B). Both TCGA and GSE156451 databases analyses showed that the expression level of LIFR-AS1 in CRC tissues was positively correlated with *LIFR* expression (Fig. 4C). The 10 CpG sites were also negatively correlated with the expression of *LIFR* (correlation coefficients from − 0.020 to − 0.385, Fig. 4D). However, overexpression of lncRNA LIFR-AS1 did not affect the mRNA level of *LIFR* in colon cancer cells (Fig. 4E). Analysis of scRNA-seq data revealed that *LIFR* was markedly increased in stromal cells and its expression was apparent in epithelial cells (Fig. 4F).

LIFR-AS1 also harbors a microRNA, miR-3650, that is transcribed in an antisense orientation. We speculated whether miR-3650 suppressed LIFR; whereas miR-3650 was not detected in CRC (Fig. 4G). Based on the differential analysis by the Wilcoxon test, we identified 517 miRNAs (202 upregulated miRNAs and 315 downregulated miRNAs) as significantly differentially expressed in CRC tissue compared with the normal samples in TCGA database (Additional file 4: Fig. S3). Then the downstream upregulated miRNAs targeted by LIFR-AS1 in CRC were determined using StarBase [19]. Correlation analysis showed that the expression levels of six target miRNAs (hsa-miR-30b-5p, hsa-miR-30e-5p, hsa-miR-4677-3p, hsa-miR-374b-5p, hsa-miR-29b-3p and hsa-miR-144-3p) were negatively correlated with LIFR-AS1 (Fig. 4H, Additional file 5: Fig. S4). We selected the most significantly associated miRNA (hsa-miR-29b-3p) into the further study. As expected, hsa-miR-29b-3p mimic prominently decreased luciferase activity in LIFR-AS1_WT group in comparison with LIFR-AS1_MUT group in both HT-29 and T84 cell lines, which indicated that hsa-miR-29b-3p is a target of lncRNA LIFR-AS1 (Fig. 4I).

### Validation of the hypermethylation status for LIFR-AS1

**Fig. 5:**
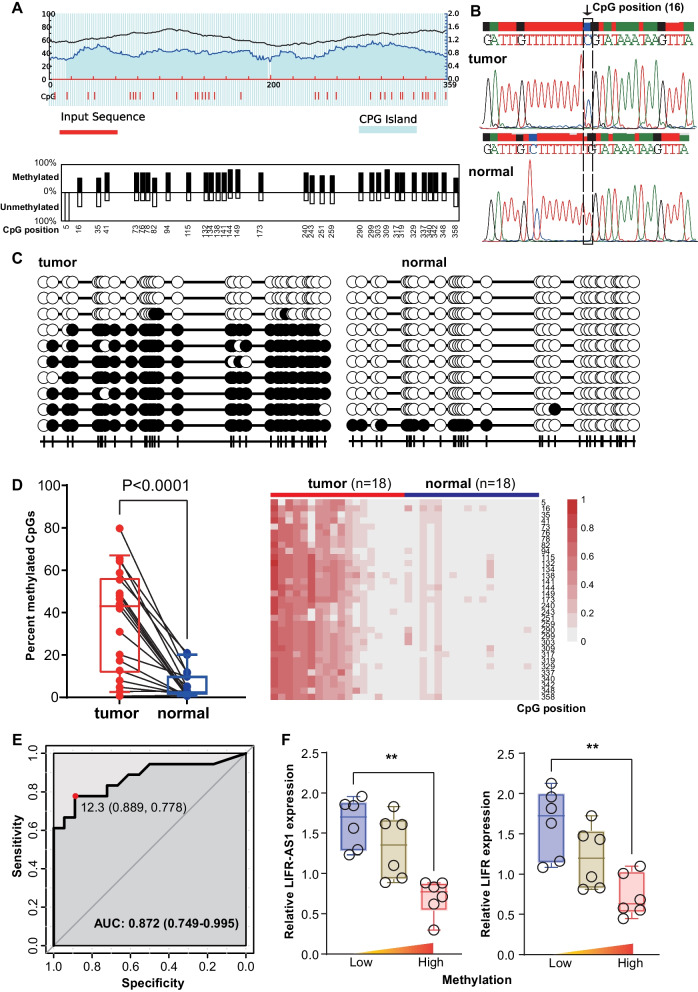
The LIFR-AS1 promoter region is hypermethylated in CRC. **A** MethPrimer predicted potential CpG islands (blue region) and depicted CpG sites (red vertical line and bar chart) within the LIFR-AS1 promoter region. **B** Bisulfite sequencing PCR (BSP) showed that a CpG site at position 16 was methylated in tumors. **C** BSP was used to detect the methylation status of LIFR-AS1 in tumors and paired adjacent normal tissues. **D** Results of BSP detection of the methylation status of the LIFR-AS1 promoter region using 18 colorectal cancers and paired adjacent normal tissues. **E** Receiver operating characteristic curve showing the profiles of sensitivity and specificity of the methylation status to distinguish colorectal cancers from normal tissues. **F** LIFR-AS1 and *LIFR* mRNA expression levels were significantly lower in hypermethylated groups compared to hypomethylated groups

The CpG island and 33 CpG sites were also predicted by MethPrimer (Fig. 5A). Therefore, the BSP approach was applied to determine the methylation status of the LIFR-AS1 promoter region in 18 colorectal tumor tissues and adjacent normal tissues (Fig. 5B, C). We observed markedly elevated average DNA methylation levels with a mean of 36.1% in CRC specimens compared with 5.5% in paired adjacent normal tissues. Consistent with our bioinformatic analysis, the LIFR-AS1 promoter region was significantly hypermethylated (*P* < 0.001, Fig. 5D). To determine the diagnostic potential of LIFR-AS1 promoter methylation status, ROC curve analysis was performed. The optimal cut-off of LIFR-AS1 promoter methylation (12.3%) was defined by maximizing the Youden index (sensitivity + specificity − 1). This cut-off discriminated between CRC and normal tissues with a sensitivity of 77.8%, a specificity of 88.9%, and an area under the curve (AUC) value of 0.872 (Fig. 5E). Moreover, we found a potential correlation between DNA methylation in the promoter region of LIFR-AS1 and *LIFR* with their mRNA expression, respectively (Fig. 5F).

### LIFR-AS1 is clinically relevant in CRC

**Fig. 6:**
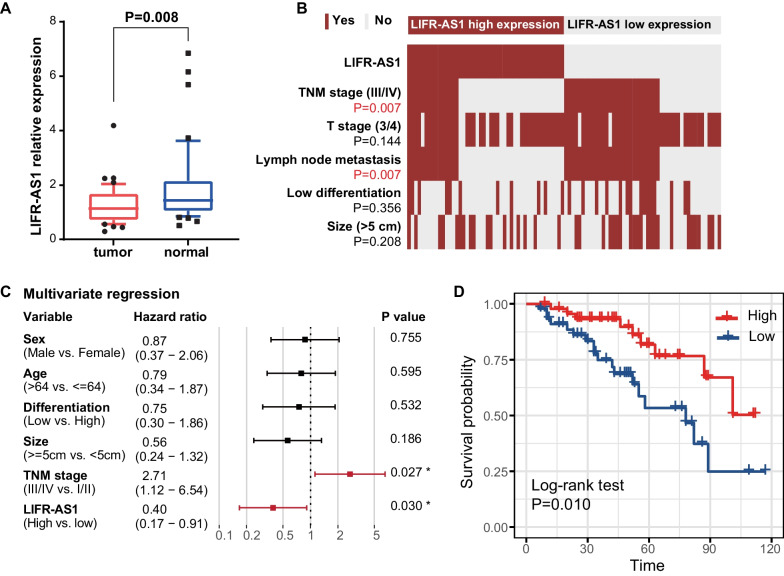
LIFR-AS1 is clinically relevant in CRC. **A** Statistical analysis of LIFR-AS1 expression in CRC and normal tissues. **B** Comparison of TNM stage, T stage, lymph node metastasis, size and histologic differentiation between the LIFR-AS1 high expression and low expression groups. **C** Multivariate Cox regression analysis of variables associated with the survival in CRC patients. **D** Kaplan–Meier analysis of overall survival for CRC patients according to LIFR-AS1 expression

We measured LIFR-AS1 expression in our cohort of 43 tumor and paired adjacent normal tissues using RT-qPCR. The 2 − ^ΔΔ^CT method was used to calculate FC for LIFR-AS1 compared with the internal control GAPDH mRNA. LIFR-AS1 was downregulated in CRC compared with normal tissues (FC = − 1.50, *P* = 0.008, Fig. 6A). We also found that LIFR-AS1 expression positively correlated with TNM stage and lymph node metastasis in 92 CRC patients (Fig. 6B). Moreover, forest plot from multivariate regression analysis demonstrated that high LIFR-AS1 related to increased overall survival (*P* < 0.05, Fig. 6C). Kaplan–Meier analysis revealed poor survival for patients with low expression of LIFR-AS1 compared with patients with high expression of LIFR-AS1 (*P* = 0.010, Fig. 6D). Thus, these findings suggest that LIFR-AS1 might be an independent predictor of CRC aggressiveness.

### LIFR-AS1 is a novel tumor suppressor lncRNA in CRC

**Fig. 7:**
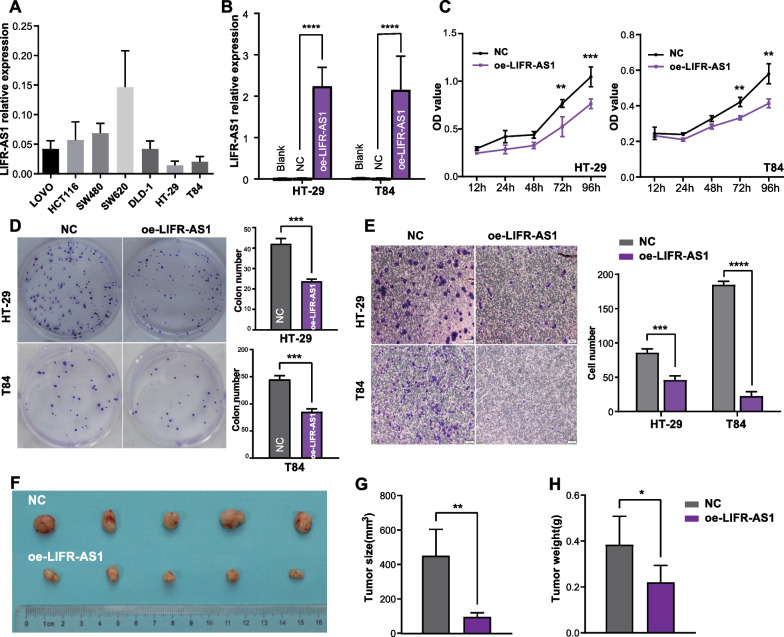
LIFR-AS1 is a tumor suppressor in CRC. **A** RT-qPCR analysis of the expression of LIFR-AS1 in colon cancer cells. **B** LIFR-AS1 expression in HT-29 and T84 cells overexpressing LIFR-AS1 or control was measured by RT-qPCR. **C** Cell viabilities of HT-29 and T84 cells overexpressing LIFR-AS1 or control were determined by CCK-8 assay. **D** Colony formation assay of HT-29 and T84 cells overexpressing LIFR-AS1 or control. **E** Transwell Matrigel invasion assay was performed in HT-29 and T84 cells transfected with LIFR-AS1 or control. **F** Representative data of tumors in nude mice derived from colon cancer cells transfected LIFR-AS1 or control. **G** Tumor volume of the LIFR-AS1 and negative control groups. **H** Tumor weight of the LIFR-AS1 and negative control groups (oe-LIFR-AS1: HT-29 or T84 cells transfected with LIFR-AS1 expression plasmid; NC: HT-29 or T84 cells transfected with the empty vector)

We next investigated the potential roles of LIFR-AS1 in CRC in vitro and in vivo. RT-qPCR demonstrated that the expression of LIFR-AS1 in colon cancer cells (HT-29 and T84) was appreciably lower than that in other cells (LOVO, HCT116, SW480, SW620 and DLD-1) (Fig. 7A). We thus chose HT-29 and T84 cells for further analysis and overexpressed LIFR-AS1 using oe-LIFR-AS1 (Fig. 7B). Both HT-29 and T84 cells overexpressing LIFR-AS1 displayed lower cell viabilities than the respective control cells (Fig. 7C). Stable overexpression of LIFR-AS1 induced a significant decrease in colony formation and cell invasion (Fig. 7D, E). To confirm the above observed phenotype, xenograft mouse models were established. The results showed that overexpression in LIFR-AS1 cells decreased the average volume and weight of tumors (Fig. 7F–H). Together, these findings indicate that LIFR-AS1 functions as a tumor suppressor in CRC.

## Discussion

Recent multi-omics analysis has demonstrated that cancer involves a complex regulatory network that harbors both genetic and epigenetic abnormalities, contributing to escape from chemotherapy and host immune surveillance [20, 21]. Advances in high-throughput sequencing technologies have led to the identification of individual molecular heterogeneity. Epigenetics including DNA methylation and histone modifications are a new research focus in cancer [22]. Methylation features were found to be closely linked to CRC patient prognosis. For example, methylation levels in the intragenic regions of oncogenes (*PDX1*, *EN2*, and *MSX1*) levels could predict CRC patient prognosis [23]. In this study, we performed an integrated methylome and transcriptome analysis to identify potential lncRNAs regulated by aberrant DNA methylation in CRC. By precisely mapping altered DNA methylation to the promoter regions of lncRNAs, 276 epigenetically deregulated lncRNAs in CRC were identified. To confirm the accuracy of the approach, BSP assay was performed, and the results showed high methylation status of LIFR-AS1 promoter region. Four CpG sites (cg12587766, cg18174928, cg12602374 and cg18848688) and the expression of LIFR-AS1 were related to the prognosis of CRC. Overexpression of LIFR-AS1 was shown to remarkably suppress colon cancer cell proliferation, growth and invasion in vitro and in vivo.

LIFR-AS1 is located on chromosome 5p13.1 and transcribed in an antisense manner from the *LIFR* gene. Several human solid tumors have been shown to exhibit aberrant expression of LIFR-AS1 [24–26]. Liu et al. found that LIFR-AS1 and miR-29a negatively regulated each other through direct binding in PDT-treated HCT116 cells [24]. LIFR-AS1 knockdown reduced the effect of PDT on proliferation and apoptosis of CRC cells, implying that LIFR-AS1 may act as a tumor suppressor via interacting with miR-29a. Pan et al. observed that the expression levels of LIFR-AS1 were significantly increased in gastric tumor tissues and cells compared with normal adjacent tissue samples and GSE1 cells [26]. LIFR-AS1 modulates *COL1A2* to promote gastric cancer cell proliferation and migration by miR-29a-3p. Chen et al. reported that METTL3-mediated m^6^A hyper-methylation induced the upregulation of LIFR-AS1 in pancreatic cancer by enhancing *METTL3* mRNA stability, resulting in increased expression of *VEGFA* by directly interacting with miR-150-5p [25]. LIFR-AS1 has been shown to act as a sponge for miR-942-5p in lung cancer [27], for miR-29a in CRC [24] and osteosarcoma [28], for miR-31-5p in thyroid carcinoma [29], for miR-4262 in glioma [30], for miRNA-150-5p in pancreatic cancer [25], for miR-197-3p in breast cancer [31], and for miR-29a-3p [26] and miR-4698 [32] in gastric cancer. These results prompted us to investigate the ceRNA function of LIFR-AS in CRC. Interestingly, we found that LIFR-AS1 could interact with hsa-miR-29b-3p through luciferase reporter gene in colon cancer cells.

Considering the heterogeneity of LIFR-AS1 in cancers, we performed scRNA-seq on CRC and non-tumor cells of the single-cell expression to characterize the functional role of LFIR-AS1 indirectly. Intriguing, *SNRPF*, which is negatively linked to LIFR-AS1, was highly expressed in CRC cells. A previous study showed that *SNRPN* was highly expressed in CRC tissues and high *SNRPN* expression indicated a poor prognosis [33]. Additionally, the GSEA-mined “Genes downregulated in colorectal adenoma compared to normal mucosa samples” gene set was related to LIFR-AS1. Colorectal adenomas are often precursor lesions of CRC. These phenomena suggested LIFR-AS1 is an important tumor-suppressive lncRNA during carcinogenesis. A strong association was observed between LIFR-AS1 and *LIFR* in CRC, whereas *LIFR* expression was not detected in epithelial cells. Rockman et al. demonstrated that colonic epithelial cells express LIF protein but not LIFR. Conversely, pericryptal fibroblasts express LIFR but not LIF protein [34]. This is consistent with the results of scRNA-analysis in detecting LIFR expression in stromal cells. Meanwhile, overexpression of LIFR-AS1 did not affect the expression of *LIFR* in colon cancer cells. Differences in tissue distribution led to little biological correlation between LIFR-AS1 and *LIFR*. As a result, the displayed relationship between LIFR-AS1 and *LIFR* might due to the methylation status in the promoter region.

Methylation of cytosines in the human genome is a critical epigenetic modification that functions in transcriptional silencing [35]. DNA methylation regulates tissue-specific gene expression [36]. However, the genome-wide identification of abnormal DNA methylation in a specific lncRNA region with functional importance is lacking. In this study, we found hypermethylation of a CpG island located in the promoter region of the tumor suppressor gene LIFR-AS1 that enhanced cancer progression. The methylation level of LIFR-AS1 had high sensitivity and specificity for the diagnosis of CRC. Recent studies indicate that aberrant DNA methylation is an early and frequent event in carcinogenesis [37]. We found that early CRC tumors had high levels of LIFR-AS1 methylation, and four CpG sites were associated with prognosis. Detection of the methylation level of LIFR-AS1 might be a promising biomarker for CRC screening.

Combining bioinformatic analysis and experimental verification, our research highlighted the role of LIFR-AS1 in the progression of CRC. However, our study has several limitations. First, conjoint analysis identified a series of methylation-driven lncRNAs, and we only chose LIFR-AS1 for validation. Second, LIFR-AS1 appeared to play a significant role in determining the developing of colon cancer both in vitro and in vivo, and recent studies reported that LIFR-AS1 could interact with other molecules in multiple cancers. Thus, LIFR-AS1 may be involved in other cancers in addition to CRC. Third, whether the methylation status of LIFR-AS1 functions as an independent diagnostic and prognostic marker for CRC remains to be investigated and requires clinical multicenter studies with larger samples to confirm our findings. Fourth, the FC in LIFR-AS1 overexpression experiments was much higher than that in RNA-seq or our validation cohort and might not reflect changes in the human body.

In conclusion, by using integrative analysis and molecular experiments, we revealed that the promoter region of LIFR-AS1 was hypermethylated in CRC and was negatively associated with the expression of LIFR-AS1. Furthermore, LIFR-AS1 was correlated with poor outcome of CRC patients and repressed tumor cell growth and metastasis. Overall, our results indicate that aberrant DNA methylation mediates downregulation of LIFR-AS1 to promote the progression of colon cancer.

## Supplementary Information

**Additional file 1: Figure S1**. Pie chart shows the number of differentially expressed lncRNAs in each category.

**Additional file 2: Table S1**. The characteristics of methylation-driven lncRNAs.

**Additional file 3: Figure S2**. Correlation (*P* values derive from Spearman’s correlation) between DNA methylation and the expression of LIFR-AS1 in CRC samples.

**Additional file 4: Figure S3**. Volcano plot showing the log2 fold change of 517 significantly differentially expressed miRNAs in CRC patients from the TCGA database.

**Additional file 5: Figure S4**. Prediction of 6 miRNAs (miR-29b-3p, miR-4677-3p, miR-144-3p, miR-30b-5p, miR-30e-5p and miR-374b-5p) targeting LIFR-AS1 in CRC.

## Data Availability

All data generated or analyzed during this study are included in this article. The level 3 TCGA data for DNA methylation arrays and lncRNA expression are available in Xena website. HTSeq-Counts: https://gdc-hub.s3.us-east-1.amazonaws.com/download/TCGA-COAD.htseq_counts.tsv.gz. HTSeq-FPKM-UQ: https://gdc-hub.s3.us-east-1.amazonaws.com/download/TCGA-COAD.htseq_fpkm-uq.tsv.gz. HumanMethylation450: https://gdc-hub.s3.us-east-1.amazonaws.com/download/TCGA-COAD.methylation450.tsv.gz. The level 3 miRNA-seq data analyzed in this study are available at Genomic Data Commons portal (https://portal.gdc.cancer.gov/). The bulk RNA-seq and scRNA-seq data that support this study are available through the NCBI database under accession GSE156451 and GSE144735, respectively.
