# Supplementary figures and images for: Genome-wide screening for differentially methylated long noncoding RNAs identifies LIFR-AS1 as an epigenetically regulated lncRNA that inhibits the progression of colorectal cancer
Source: Clin Epigenetics. 2022 Oct 31;14:138. doi: 10.1186/s13148-022-01361-0 (PMC9624034; doi:10.1186/s13148-022-01361-0)

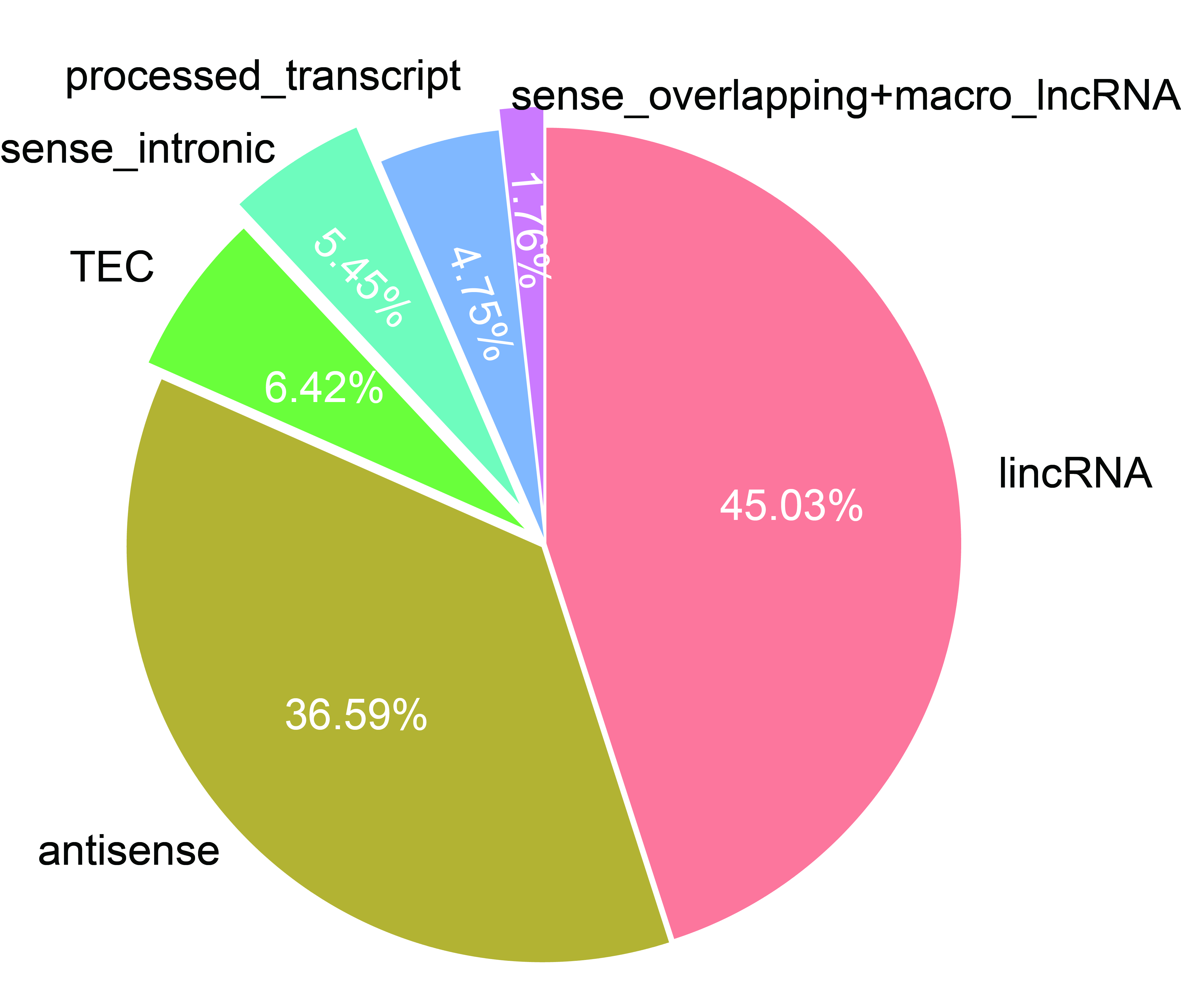

Supplement: Supplementary file 1 — Additional file 1: Figure S1. Pie chart shows the number of differentially expressed lncRNAs in each category. [file 13148_2022_1361_MOESM1_ESM.jpg]

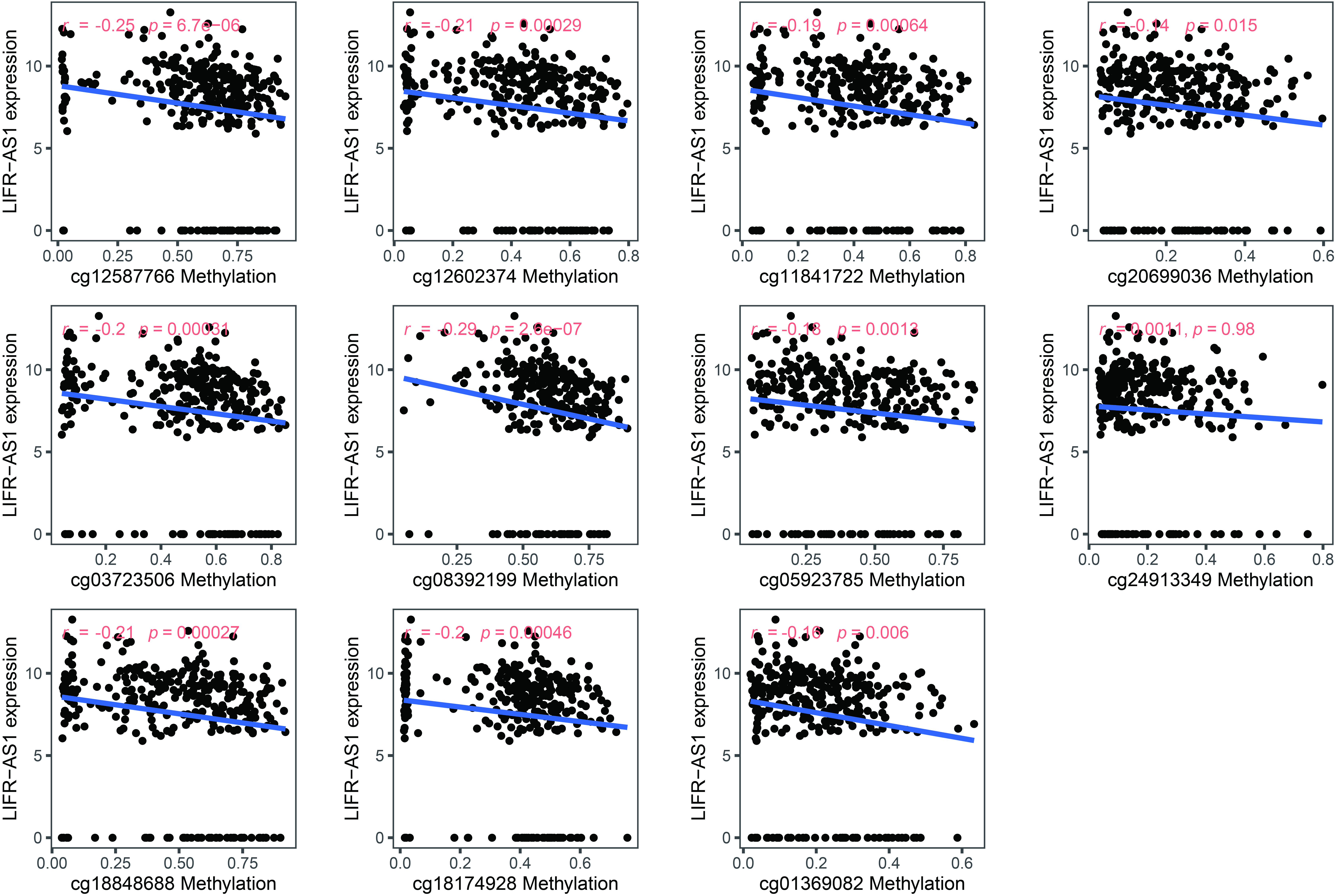

Supplement: Supplementary file 3 — Additional file 3: Figure S2. Correlation (P values derive from Spearman’s correlation) between DNA methylation and the expression of LIFR-AS1 in CRC samples. [file 13148_2022_1361_MOESM3_ESM.jpg]

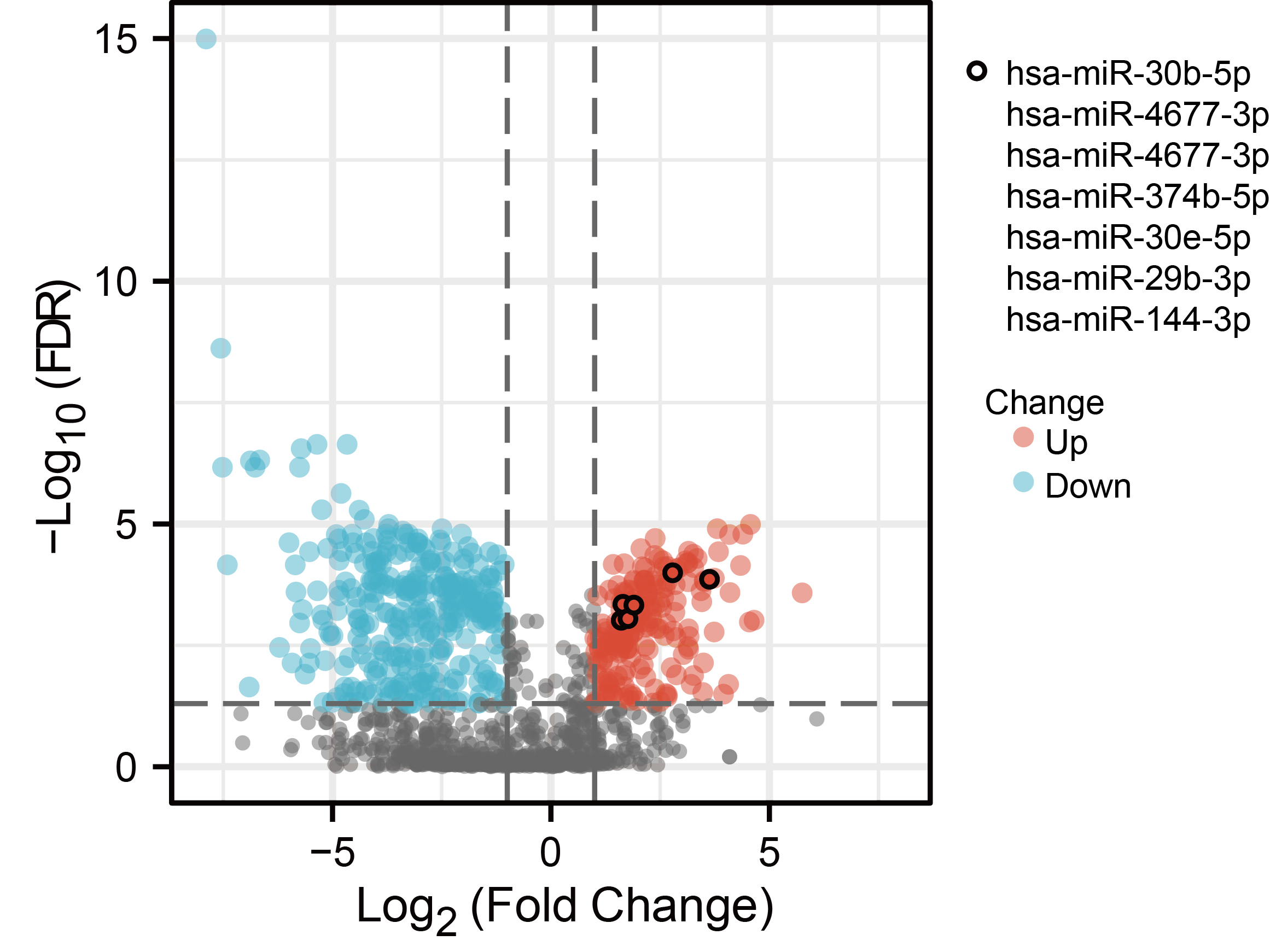

Supplement: Supplementary file 4 — Additional file 4: Figure S3. Volcano plot showing the log2 fold change of 517 significantly differentially expressed miRNAs in CRC patients from the TCGA database. [file 13148_2022_1361_MOESM4_ESM.jpg]

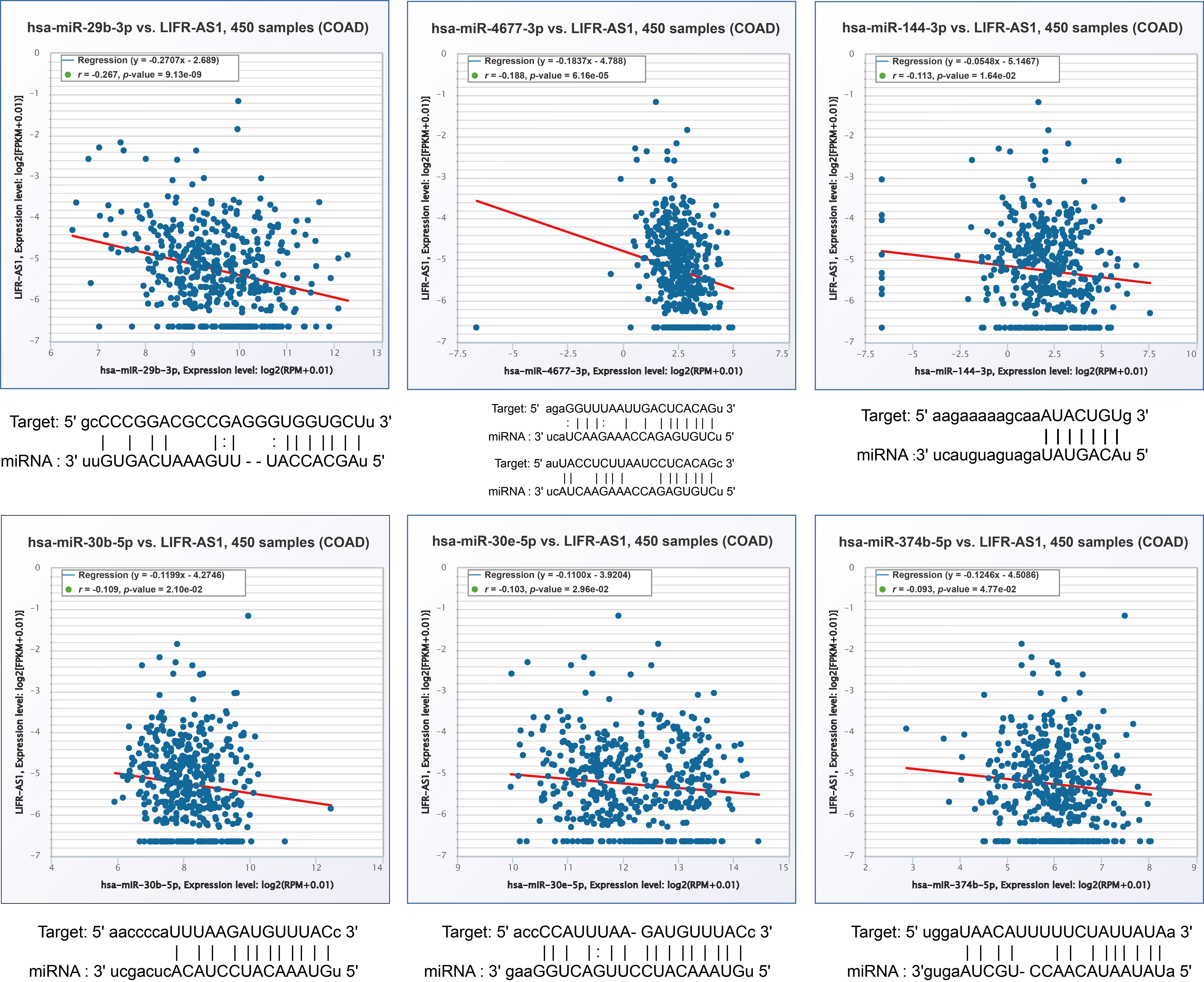

Supplement: Supplementary file 5 — Additional file 5: Figure S4. Prediction of 6 miRNAs (miR-29b-3p, miR-4677-3p, miR-144-3p, miR-30b-5p, miR-30e-5p and miR-374b-5p) targeting LIFR-AS1 in CRC. [file 13148_2022_1361_MOESM5_ESM.jpg]
